# Is quality of life after mastectomy comparable to that after breast conservation surgery? A 5-year follow up study from Mumbai, India

**DOI:** 10.1007/s11136-019-02351-1

**Published:** 2019-11-11

**Authors:** K. V. Deepa, A. Gadgil, Jenny Löfgren, S. Mehare, Prashant Bhandarkar, N. Roy

**Affiliations:** 1 Department of Surgery, Manipal Hospital, New Delhi, India; 2 Department of Surgery, BARC Hospital, WHO Collaboration Center for Research in Surgical Care Delivery in LMIC, Mumbai, India; 3 Department of Molecular Medicine and Surgery, Karolinska Institute, Stockholm, Sweden; 4 WHO Collaboration Center for Research in Surgical Care Delivery in LMIC, Mumbai, India; 5 BARC Hospital, WHO Collaboration Center for Research in Surgical Care Delivery in LMIC, Mumbai, India; 6 Public Health Systems, Karolinska Institute, Stockholm, Sweden

**Keywords:** Quality of life, Mastectomy, Breast conserving surgery, Post surgery quality of life

## Abstract

**Purpose:**

Breast cancer is the commonest cancer in women worldwide. Surgery is a central part of the treatment. Modified radical mastectomy (MRM) is often replaced by breast conserving therapy (BCT) in high-income countries. MRM is still the standard choice, in low- and middle-income countries (LMICs) as radiotherapy, a mandatory component of BCT is not widely available. It is important to understand whether quality of life (QOL) after MRM is comparable to that after BCT. This has not been studied well in LMICs. We present, 5-year follow-up of QOL scores in breast cancer patients from India.

**Methods:**

We interviewed women undergoing breast cancer surgery preoperatively, at 6 months after surgery, and at 1 year and 5 years, postoperatively. QOL scores were evaluated using FACT B questionnaire. Average QOL scores of women undergoing BCT were compared with those undergoing MRM. Total scores, domain scores and trends of scores over time were analyzed.

**Results:**

We interviewed 54 women with a mean age of 53 years (SD 9 ± years). QOL scores in all the women, dipped during the treatment period, in all subscales but improved thereafter and even surpassed the baseline in physical, emotional and breast-specific domains (*p* < 0.05) at 5 years. At the end of 5 years, there was no statistically significant difference between the MRM and BCT groups in any of the total or domain scores.

**Conclusion:**

QOL scores in Indian women did not differ significantly between MRM and BCT in the long term. Both options are acceptable in the study setting.

## Introduction

With two million new cases and over half a million deaths each year, breast cancer is the most common cancer in women worldwide [1]. Advanced stage at presentation and the lack of timely access to high-quality, affordable cancer treatment remain key barriers to improved cancer survival in limited resource settings such as India [2].

Surgery is central in breast cancer treatment and two main options for breast cancer surgery exist; mastectomy and breast conserving surgery. Mastectomy with axillary dissection is a much extensive surgery compared to conservation of breast with sentinel lymph node biopsy practiced in high-income countries (HICs). Breast conserving surgery together with postoperative radiation therapy shows similar results in terms of survival to mastectomy [3, 4]. Several prospective long-term studies on quality of life (QOL) after breast surgery carried out in HICs have shown that body image-related QOL scores are better in the patients undergoing breast conservation surgery than in those who undergo mastectomy. Mastectomy being a much extensive surgery is expected to result in poorer QOL [5, 6]. Consequently, breast conservation surgery is promoted in HICs.

Breast cancer remains first ranked cancer in women in India, with 160,000 new cases and 87,000 deaths per year [7]. Mastectomy remains the mainstay of breast cancer surgery in India as well as many other Asian and African countries, where access to radiotherapy (RT) is extremely limited and the training in breast conservation surgery has not been widely implemented [8, 9]. It has been well documented that the choice of surgical treatment modality does not affect the survival in the long term, but similar  studies comparing QOL between modified radical mastectomy (MRM) and breast conserving therapy (BCT) are few from low- and middle-income countries (LMICs). In addition, there is a lack of information about trends in QOL over time in breast cancer patients following surgery [10, 11].

The present study investigated time trends in QOL up to 5 years postoperatively after breast conserving surgery versus mastectomy in an Indian population. This information can be used to tailor the treatment suitable to available expertise and resources in this low-resource setting.

## Methods

### Study design: prospective cohort study

#### Study setting

India, a lower middle-income country, is the world’s second-most populated country with a population of 1.35 billion [12]. The present study was conducted in a secondary level community hospital at Mumbai, a metropolis city with 22.8 million inhabitants [13]. The study hospital caters to a population of 100,000 middle socioeconomic class people. These are employees and their families, within a Department of the Government of India and are covered by a comprehensive insurance scheme through which they are entitled to lifelong free and equitable healthcare. There are 15 community dispensaries spread across the city and patients are referred to a central referral 390-bed hospital (BARC Hospital), where the diagnosis and treatment for breast cancer is carried out. The electronic medical records (EMRs) makes it possible to retrieve the data on demography as well as investigations and clinical notes for follow-up of the patients [14].

#### Patients

One hundred consecutive patients with operable breast cancer, who underwent surgery as the first modality of treatment, at BARC from 2001 to 2009, were included in the study. Patients needing neoadjuvant chemotherapy (CT) and metastatic cancers were excluded. Patients who developed local relapse or metastases during the follow-up period were also excluded from all data analyses as this would affect QOL scores. The cohort of patients whose ‘one-year QOL scores’ were calculated up to year 2010 (published elsewhere) [15], was followed up prospectively for 5 years and those patients were recruited in this study.

#### Follow-up

All study participants attended a dedicated breast cancer clinic for follow-up after the operation. The protocol stipulates a visit once every 3 months for 1 year, followed by once every 6 months for 4 years and once yearly, thereafter. The patients were requested to fill the questionnaire for the QOL scores during these follow-up visits to the breast clinics. Informed consent was taken from each patient and from patients’ attendant family member in case of illiterate women.

#### Data collection

All the patients were clinically suitable for either surgical options (Table 1 cohort characteristics).

**Table 1:** Characteristics of the cohort

Characteristic	MRM	BCT	*p*-value
Number of patients (row%)	28 (51.8%)	26 (48.2%)	
Age at surgery	51.9 (SD ± 7.9)	59.1 (SD ± 8.8)	
Education status			
English educated	14 (50%)	14 (53.8%)	0.835
Could not read or write English (vernacular + illiterate)	7 (25%)	8 (30.7%)	
No data	7 (25%)	4 (15.3%)	
Marital status			
Married	28 (100%)	25 (96.1%)	0.000*
Unmarried	0 (0%)	1 (3.8%)	
Menopausal status			
Premenopausal ()	3 (10.7%)	9 (34.6%)	0.035*
Postmenopausal	25 (89.2%)	17 (65.3%)	
Stage at diagnosis			
I	4 (14.2%)	4 (15.3%)	0.807
II	19 (67.8%)	19 (73%)	
III	5 (17.8%)	3 (11.5%)	
Adjuvant chemo (*n* = 38, *p* = 0.3)			
Y	18 (64.2%)	20 (76.9%)	0.300
N	10 (35.7%)	6 (23%)	
Adjuvant radiotherapy (*n* = 31, *p* = 0.000)	6 (21.4%)	26 (100%)	
Patients survival during follow up period			
Died	17 (25.7%)	2 (5.8%)	0.016*
Survived	49 (74.3%)	32 (94.2%)	

*p < 0.05, **p < 0.001

Data were collected using questionnaires at four different points in the treatment and follow-up; preoperatively on admission to the ward, 6 months after surgery, 1 year after surgery and at 5 years after surgery. Patients were requested to fill the questionnaires and return them to the team. Patients who were unable to read and write English were interviewed by members of data collection team in Marathi/Hindi, the language spoken in the region. The interviewers were trained to make sure that questions are not suggestive or leading to a particular response. The data contained QOL scores, demographic factors of age, sex, educational level, partner status and menstrual status at the time of diagnosis. Details of the oncologic factors and histological features of the tumor were also collected.

A written informed consent was obtained from all the participants. They were informed about the option of undergoing mastectomy or breast conserving surgery and also that the latter would necessitate RT. The patients were then offered to select operation method based on their preference, followed by a uniform and standard treatment protocol for RT and CT.

#### Instrument

‘The Functional Assessment of Cancer Therapy-Breast, Version 4 (FACT-B)’ validated for Indian women was used for scoring the QOL in these patients [16]. FACT-B is a 36-item scale containing 4 general subscales or domains, relating to physical symptoms (PWB), social life (SWB), functional ability (FWB) and emotional issues (EWB). The fifth subscale, the breast subscale score (BCS) considers body image, sexual satisfaction and attractiveness perceived by the patient. This contains 9 items and is specific for breast cancer (15). Higher scores indicate better QOL in all the domains.

### Data analysis

Data were documented in Microsoft Excel 2013 spreadsheet. The total scores, FACT B Trial outcome index, FACT G and FACT B total scores were calculated. ‘FACT G (global) score’ includes all the domain scores but not the breast specific concerns (BCS), ‘FACT B total score’ included all the five domain scores mentioned in the above section, FACT B trial outcome index considers functional, physical and breast domain scores. Scores were calculated as per the instructions for version 4 of the Functional assessment of Chronic Illness Therapy (FACIT) Measurement system [16]. Missing data was retrieved from case files for demographic and oncological variables.

Age was treated as continuous variable. A two-tailed Students *t* test was used to detect statistically significant differences between the two study groups. Differences in the average scores were tested using the paired *t* test. The changes (decline/increase) in the scores in all domains, over time during the study period, were analyzed separately for each group as well by combining both groups together. Values < 0.05 were considered statistically significant. Statistical Package for the Social Sciences (SPSS 24.0, IBM Corp., Armonk, NY) software package for windows was used for all analysis.

### Ethical considerations

The study was approved by the Institutional Ethics Committee (BHMEt BARC Hospital, where the study was conducted (C/DNB/14/10).

## Results

**Fig. 1:**
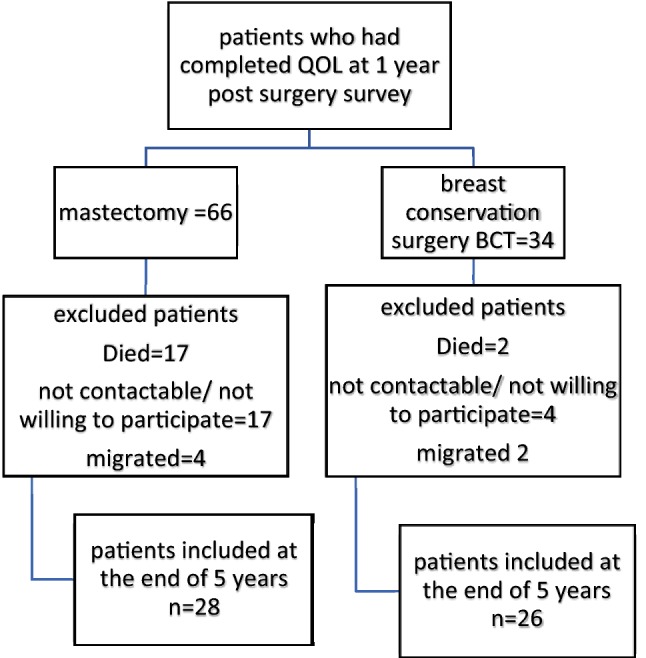
Description of cohort

Of the 100 patients included into the study, 54 were followed up after 5 years (Fig. 1 description of cohort). Table 1 describes the characteristics of the cohort. Mean age at diagnosis of the patients followed up after 5 years was 55.3 years (SD ± 9). Of the patients who were followed up after 5 years, 28 underwent MRM and 26 underwent breast conservation surgery (BCT). Two thirds of the patients in each group had breast cancer stage II.

All patients, who underwent BCT, received radiation to the breast and axilla according to Protocol of Referral Tertiary Cancer Center, Tata Memorial Hospital in Mumbai. In the MRM group, six patients received RT to the chest wall and axilla.

### QOL scores

All the scores were calculated for patients who could complete 5-year follow-up. Patients who died between the period of 1-year follow-up and 5-year follow-up were excluded from all analyses. The QOL scores for both groups in physical well-being, functional well-being and breast specific domain as well as total scores reduced significantly (*p* < 0.01) at 6 months compared to preoperatively. The scores were unchanged for social as well as emotional domains in both the groups at the end of 6 months.

The scores nearly normalized at the 1-year follow-up.

**Fig. 2:**
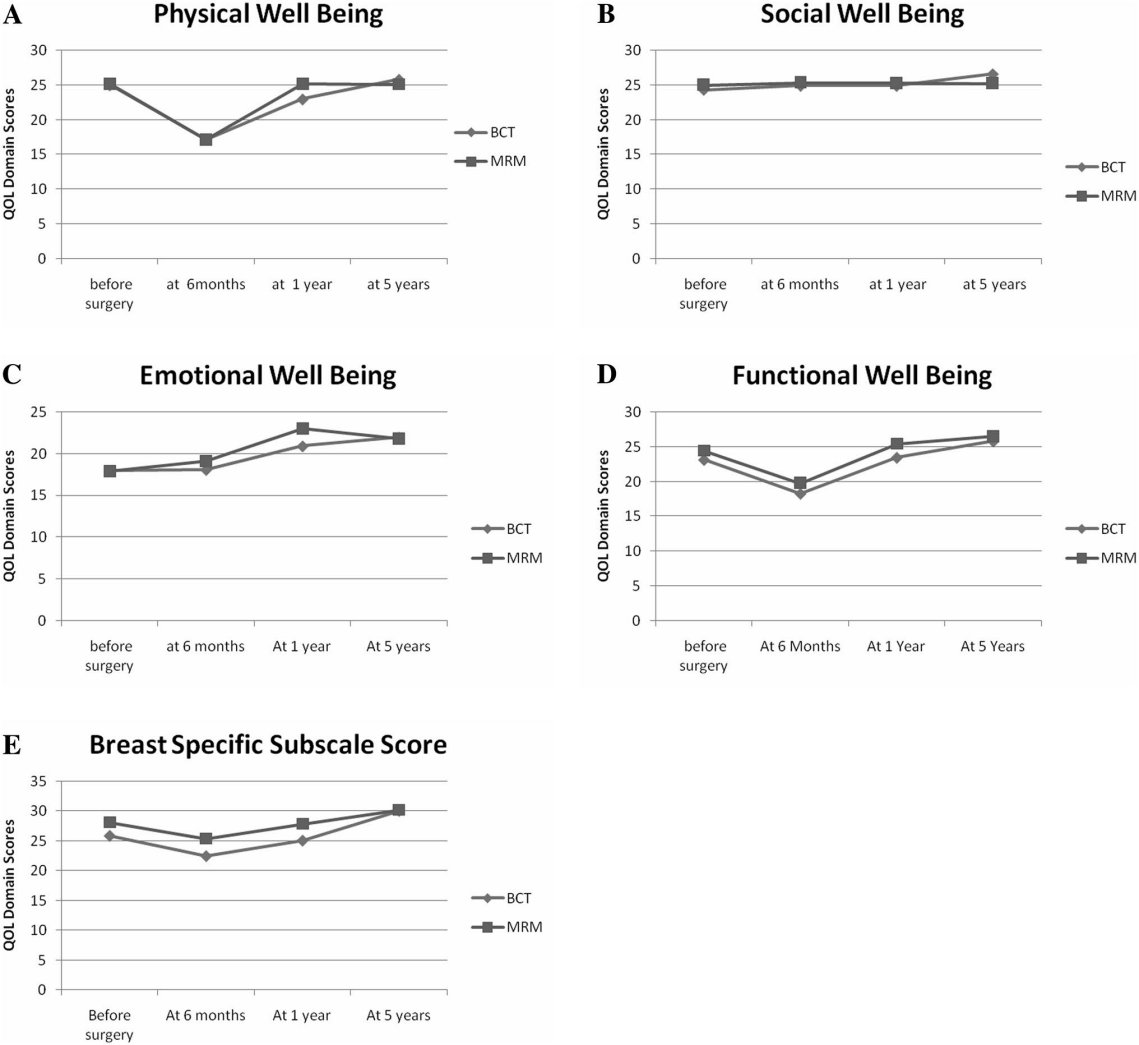
Trends in QOL scores over a period of 5 years. **a** Physical well-being scores (addresses pain, other physical symptoms, side effects of treatment). Scores in both groups were significantly lower at 6 months. MRM scores were higher than BCT group at the end of 1 year *p* = 0.019*. **b** Social Well-Being scores (addresses closeness to family, partner and support from friends). **c** Emotional Well-Being (addresses sadness, loss of hope, fear of dying). Scores at 1 year were significantly higher in MRM group at the end of 1 year, *p* = 0.014*. **d** Functional Well-being (addresses work at home, workplace, accepted illness). Scores in both groups were significantly lower at the end of 6 months. *p* = 0.047*. **e** Breast Cancer Subscale scores (body image, hair loss, change in image and look). Scores were significantly lower in both groups at the end of 6 months. Scores at 1 year were significantly higher in MRM group at the end of 1 year *p* = 0.020*

Domain scores of emotional, functional and breast specific domain, along with the total scores, had increased beyond the level of the preoperative scores (*p* < 0.05) at the end of 5 years (Table 2). Figure 2 (trends in QOL scores over a period of 5 years) demonstrates the trends in the scores over period of time.

**Table 2:** Comparison of scores between “before surgery” and “at the end of five years”, mean ± SD

Domains (maximum scores)	BCT			MRM			All combined		
	Before surgery, ± SD	Five years, ± SD	*p*-value	Before surgery, ± SD	Five years, ± SD	*p*-value	Before surgery, ± SD	Five years, ± SD	*p*-value
Physical Well-Being (28)	25.1 ± 2.7	25.8 ± 3.1	0.441	25.1 ± 3.1	25.1 ± 3.7	0.973	25.1 ± 2.9	25.4 ± 3.4	0.653
Social Well-Being (28)	24.3 ± 5.3	26.6 ± 2.7	0.060	25 ± 4.3	25.2 ± 5.2	0.839	24.7 ± 4.8	25.9 ± 4.2	0.136
Emotional Well-Being (24)	18 ± 6.9	22 ± 2.4	0.005*	17.9 ± 5.8	21.8 ± 2.9	0.001*	17.9 ± 6.3	21.9 ± 2.7	0.000*
Functional Well-Being (28)	23.2 ± 4.5	25.8 ± 2.7	0.009*	24.4 ± 4.2	26.5 ± 2	0.047*	23.8 ± 4.4	26.2 ± 2.4	0.001*
Breast Cancer Subscale (36)	25.9 ± 4.5	30.1 ± 5.4	0.007*	28.1 ± 4.6	30.2 ± 4.2	0.123	27.1 ± 4.6	30.1 ± 4.8	0.002*
FACT B Trial Outcome Index (92)	61.3 ± 16.6	81.3 ± 9.9	0.000*	64.2 ± 16.7	81.8 ± 7.9	0.000*	62.8 ± 16.6	81.6 ± 8.9	0.000*
FACT-Global score (108)	90.5 ± 12.3	100.2 ± 6.8	0.002*	92.5 ± 11.9	98.7 ± 9	0.027*	91.5 ± 12	99.4 ± 8	0.000*
FACT_B_Total Score (144)	103.6 ± 21.5	130.2 ± 10.7	0.000*	107.1 ± 20.2	128.9 ± 11.6	0.000*	105.4 ± 20.7	129.5 ± 11.1	0.000*

*p < 0.05, **p < 0.001

At the end of 1 year, domain scores of physical symptoms such as pain, nausea, weakness (PWB, 23 vs. 25, *p* = 0.01), emotional well-being scores (EWB, 21 vs. 23, *p* = 0.01), body image and attractiveness (BCS, 25 vs. 28, *p* = 0.02) and the global score which considers sum of emotional, physical symptoms and feeling of closeness to family and friends (FACT G 92 vs. 98, *p* = 0.01) were significantly better in MRM group as compared to the BCS group (Table 3). At 5 years, this difference had reduced. There was no statistically significant difference in any of the domain as well as total scores between patients undergoing BCT and those undergoing MRM.

**Table 3:** Comparison of scores between Breast conservation and Mastectomy groups, mean ± SD

Domain (maximum scores)	Time from surgery	BCT ± SD	MRM ± SD	BCT versus MRM *p*-values
Physical Well-Being (28)	One year	23 ± 4.2	25.2 ± 3.4	0.019*
	Five year	25.8 ± 3.1	25.1 ± 3.7	0.233
Social Well-Being (28)	One year	24.9 ± 3.6	25.2 ± 3.3	0.354
	Five year	26.6 ± 2.7	25.2 ± 5.2	0.112
Emotional Well-Being (24)	One year	20.9 ± 4.5	23 ± 1.3	0.014*
	Five year	22 ± 2.4	21.8 ± 2.9	0.424
Functional Well-Being (28)	One year	23.5 ± 4.6	25.4 ± 3.8	0.047*
	Five year	25.8 ± 2.7	26.5 ± 2	0.149
Breast Cancer Subscale (36)	One year	25.1 ± 5	27.9 ± 4.8	0.020*
	Five year	30.1 ± 5.4	30.2 ± 4.2	0.459
FACT B Trial Outcome Index (92)	One year	59.4 ± 17.1	65.2 ± 15.1	0.097
	Five year	81.3 ± 9.9	81.8 ± 7.9	0.413
FACT-Global scores (108)	One year	92.2 ± 12.3	98.9 ± 8.8	0.014*
	Five year	100.2 ± 6.8	98.7 ± 9	0.245
FACT_B_Total Scores (144)	One year	92.2 ± 20.1	98.9 ± 15.8	0.051
	Five year	130.2 ± 10.7	128.9 ± 11.6	0.328

*p < 0.05

Table 4 shows the comparison of trends. There was statistically significant decline in scores at 6 months and a significant increase thereafter up to 1 year in both BCT and MRM groups. The scores changed parallelly and similarly in both groups. None of the groups fared better in terms of increase in scores over time. This comparison between the amounts of change in the groups is shown in Table 5.

**Table 4:** Comparision of quality of life score within the groups separately and combined for (1) before and at 6 months, (2) 6 months and after 1 year and (3) after 1 and 5 year

Narration	BCT		MRM		Combined	
	*t* Statistic	*p* Value	*t* Statistic	*p* Value	*t* Statistic	*p* Value
(1) Differences in scores between ‘Before surgery’ and at ‘six months’: physical, functional well-being and total Scores dropped significantly at 6 months in both groups						
PWB	5.61	0.000**	5.22	0.000**	7.69	0.000
SWB	− 0.48	0.634	− 0.35	0.731	− 0.59	0.555
EWB	− 0.04	0.967	− 0.85	0.398	− 0.57	0.573
FWB	3.04	0.004*	2.89	0.006*	4.19	0.000
BCS	2.15	0.036*	1.88	0.066*	2.80	0.006
FACT_B_TOI	2.93	0.005*	2.74	0.008*	4.02	0.000
FACT_G	2.83	0.007*	2.76	0.008*	3.98	0.000
FACT_B_TS	2.17	0.035*	2.10	0.040*	3.04	0.003
(2) Differences in scores ‘At six months’ and ‘After 1 Year from Surgery’ physical, functional well-being and total scores increased significantly at 6 months in both groups						
PWB	− 3.76	0.000*	− 5.17	0.000**	− 6.35	0.000
SWB	0.03	0.977	0.13	0.901	0.11	0.915
EWB	− 1.92	0.061	− 4.86	0.000**	− 4.07	0.000
FWB	− 3.21	0.002*	− 3.62	0.001*	− 4.82	0.000
BCS	− 1.60	0.116	− 1.70	0.095	− 2.28	0.024
FACT_B_TOI	− 2.51	0.016*	− 3.05	0.004*	− 3.94	0.000
FACT_G	− 3.22	0.002*	− 4.68	0.000**	− 5.52	0.000
FACT_B_TS	− 2.48	0.017*	− 3.44	0.001*	− 4.15	0.000
(3) Differences in scores ‘After 1 Year’ and ‘5 Year from Surgery’; physical and breast subscale scores increased significantly after 1 year in BCT group but not in MRM group. Total scores increased further after 1 year in both groups						
PWB	− 2.77	0.008*	0.08	0.940	− 1.87	0.064
SWB	− 1.97	0.055	0.00	0.998	− 1.13	0.262
EWB	− 1.04	0.305	2.02	0.048*	0.22	0.826
FWB	− 2.23	0.030	− 1.32	0.191	− 2.53	0.013
BCS	− 3.43	0.001*	− 1.88	0.065	− 3.76	0.000
FACT_B_TOI	− 5.66	0.000**	− 5.17	0.000**	− 7.64	0.000
FACT_G	− 2.88	0.006*	0.09	0.928	− 2.00	0.048
FACT_B_TS	− 5.60	0.000**	− 4.16	0.000**	− 6.88	0.000

*p < 0.05, **p < 0.001

**Table 5:** Comparison of “change in scores” between both study group over time

Narration	Mean ± SD		*t* test for equality of means		
	BCT	MRM	*t*	*p* value	MD (95 CI)
Changes in scores between ‘Before surgery’ and ‘at six months from surgery’					
PWB	8 ± 7.2	8.04 ± 7.6	− 0.018	0.986	− 0.04 (− 4.1 to 4)
SWB	− 0.6 ± 3.2	− 0.34 ± 3.5	− 0.271	0.787	− 0.25 (− 2.1 to 1.6)
EWB	− 0.1 ± 3.3	− 1.14 ± 5.7	0.831	0.410	1.07 (− 1.5 to 3.6)
FWB	4.9 ± 6	4.64 ± 6.9	0.137	0.891	0.24 (− 3.3 to 3.8)
BCS	3.4 ± 5.4	2.7 ± 4.8	0.524	0.603	0.72 (− 2 to 3.5)
FACT_B_TOI	14.6 ± 12.5	14.11 ± 15.1	0.119	0.906	0.45 (− 7.2 to 8.1)
FACT_G	12.2 ± 11.5	11.2 ± 16.3	0.265	0.792	1.02 (− 6.7 to 8.8)
FACT_B_TS	13.9 ± 12.9	12.63 ± 17.7	0.299	0.766	1.27 (− 7.2 to 9.8)
Changes in scores between ‘At six months’ and ‘After 1 Year’					
PWB	− 5.8 ± 7.2	− 8.07 ± 6.5	1.191	0.239	2.23 (− 1.5 to 6)
SWB	0 ± 1	0.11 ± 1.8	− 0.200	0.842	− 0.08 (− 0.9 to 0.7)
EWB	− 2.9 ± 4.9	− 3.96 ± 4.3	0.864	0.391	1.08 (− 1.4 to 3.6)
FWB	− 5.2 ± 6.3	− 5.68 ± 6.7	0.274	0.785	0.49 (− 3.1 to 4)
BCS	− 2.6 ± 4.7	− 2.5 ± 4.2	− 0.099	0.921	− 0.12 (− 2.6 to 2.3)
FACT_B_TOI	− 12.6 ± 12.7	− 15.11 ± 12.8	0.718	0.476	2.5 (− 4.5 to 9.5)
FACT_G	− 13.9 ± 15.5	− 17.61 ± 15	0.894	0.376	3.71 (− 4.6 to 12)
FACT_B_TS	− 15.5 ± 16.5	− 18.97 ± 16.2	0.787	0.435	3.5 (− 5.4 to 12.4)
Changes in scores between ‘At 1 Year’ and ‘At -5 Year’					
PWB	− 2.8 ± 5.4	− 0.58 ± 4.8	− 1.617	0.112	− 2.24 (− 5 to 0.5)
SWB	− 1.7 ± 4.6	− 1.48 ± 2.6	− 0.254	0.800	− 0.26 (− 2.3 to 1.8)
EWB	− 1 ± 4.6	0.96 ± 2.7	− 1.970	0.054	− 2 (− 4 to 0)
FWB	− 2.3 ± 5.3	− 0.5 ± 4.7	− 1.360	0.180	− 1.85 (− 4.6 to 0.9)
BCS	− 5 ± 6.8	− 2.28 ± 6.9	− 1.433	0.158	− 2.68 (− 6.4 to 1.1)
FACT_B_TOI	− 21.9 ± 20.3	− 16.36 ± 18.1	− 1.057	0.295	− 5.52 (− 16 to 5)
FACT_G	− 7.9 ± 14.1	− 1.59 ± 10.7	− 1.872	0.067	− 6.34 (− 13.1 to 0.5)
FACT_B_TS	− 25 ± 22.9	− 17.23 ± 18.5	− 1.385	0.172	− 7.81 (− 19.1 to 3.5)

Both, BCT and MRM groups had similar changes in scores through the study period

## Discussion

Our study highlights the longitudinal trends in QOL over time. No statistically significant difference was found in the long-term QOL of BCT and MRM groups. Initially, QOL values were reduced during the treatment for all patients, which improved thereafter.

This study demonstrates similar QOL scores 5 years after BCT and MRM in urban community, in Mumbai.

Several studies and review articles based on research in HICs have investigated the effect of type of surgery on QOL in breast cancer patients [3, 17, 18]. These studies have mainly shown higher QOL in BCT patients compared to MRM patients, in the domains of body image [17, 19, 20] and cognitive and role functioning [21].

However, the evidence is not conclusive. In a review of RCTs comparing BCT with MRM, only four out of eight RCTs demonstrated that BCT patients had better QOL than mastectomy patients [22]. Another review found that there was no difference in the QOL between BCT and MRM groups [23]. A review by Oshumi et al. concluded that results were often unreliable due to lack of randomization [24]. A study from Taiwan showed that QOL was worse in BCT patients when compared to mastectomy patients [25]. In India, where facilities for breast conservation and mammography may not be available, BCT may not be a feasible option. Our results showing statistically similar QOL scores after BCT and MRM, in the long follow-up period, makes MRM as an effective option and hence assume significance for clinical practice, with equal QOL.

Body image and attractiveness (BCS, 25 vs. 28, *p* = 0.02), physical symptoms such as pain, nausea, and weakness (PWB, 23 vs. 25, *p* = 0.01) were better in MRM patients, in our study, at the end of 1 year. Many studies from HICs, addressing short term as well as long-term follow-up, have emphasized the better body image scores after BCT compared to MRM [17]. Recent study from India showed that 40% of patients had good and 55% had moderate body image scores after mastectomy [26]. A study comparing BCT and MRM groups from southern part of India showed BCT patients had worse global, body image and sexual function scores at 18 months from surgery [27]. They noticed overriding financial constraints due to length and expenses of treatment in patients undergoing BCT. Our study is done in an environment, where the patient’s choice and suitability of patient to undergo conservation were the only concerns to choose the treatment options. This has allowed women to participate in the decision-making and they have undergone mastectomy or conservation of breast, by choice. This could have had effect on the scores making mastectomy more acceptable to them. Since both groups had the opportunity to choose the surgery, it puts the study in an ideal setting where BCT and MRM can be evaluated and compared for QOL in Indian scenario. We did not find breast conservation to be superior to MRM. Other studies from India could not demonstrate BCT to have superior QOL [26, 27].

Reference [28]. The long-term follow-up studies in Indian context are lacking in documenting, whether that removal of the breast leads to poor QOL scores and whether body image is a major concern affecting them.

Emotional well-being scores (21 vs. 23, *p* = 0.014) and the global score which considers sum of emotional, physical symptoms and feeling of closeness to family and friends (FACT G 92 vs. 98, *p* = 0.01) were significantly better in MRM group as compared to the BCS group at the end of 1 year., but the difference diminished over the period of 5 years. The strong emotional support in family and strong religious beliefs may be a possible explanation that has played a significant role in preserving emotional well-being of women undergoing mastectomy in our study, where a stronger bond and a feeling of ‘being looked after’ in postoperative period may have decreased the effect of surgery in MRM group. A cross-sectional study from Egypt showed better global scores in mastectomy group along with body image scores. They also showed social and emotional domain scores to be similar in mastectomy as well as BCT groups. The authors have similarly explained that strong religious beliefs and cultural practices in Egypt may have helped the women to cope better with mastectomy [10]. A review of Indian literature on QOL in breast cancer patients has documented economical constraints, cultural issues, poor literacy, spirituality, distance traveled for treatment as determinants of QOL in Indian breast cancer patients [29]. These social and cultural influences in the Indian scenario need to be further evaluated by studies addressing coping strategies to cope with breast cancer and the emotional well-being in Indian women.

The QOL scores for physical well-being, functional well-being and breast-specific domain reduced at 6 months compared to preoperatively and then improved over further follow up period. This trend of dipping scores during the treatment phase can be attributed to acute side effects of surgery and adjuvant treatment. Both MRM as well as BCT groups showed this statistically significant decline and neither of the groups fared better than the other. Active treatment period of CT and RT, though reduced QOL due to their severe side effects, the emotional and social well-being scores did not show statistically significant decline. This may suggest strong family and social support in the Indian family structure that patients could have received. The scores nearly normalized at the 1-year follow-up. A previous Indian study showed that initial difference in QOL scores disappeared by the end of 18 months [28]. The QOL picture takes time to emerge and some changes become apparent at the end of 5 years [3]. This is consistent with previous research where active or recent adjuvant therapy has been found to be associated with reduced QOL [30, 31]. Similar observations were made when Okoli et al. studied QOL in Nigeria, following breast cancer treatment [20]. There was agreement in the literature that QOL scores over longer follow-up period are required for the acute effects of treatment to wean off and lasting effects to emerge [32].

Choice of surgical method should be considered in the light of expected survival outcome and impact on QOL as well as available resources and patient preference. Indian women do not have uniform access to RT which is a necessary adjuvant to BCS. If RT services are not available, clearly BCS cannot be recommended. The cost and the logistic support that women may need to complete additional RT in BCS can be considerable and in fact, distance traveled for cancer treatment therapy is predictor of QOL as demonstrated by the Indian study by Pandey et al. [16]. For the resource poor setting in India, mastectomy still has a definitive role to play for women who do not have access and resources to complete the adjuvant treatment for BCS. In a country where the survival after breast cancer is the most important issue, type of treatment does not seem to impact QOL Additional supports from cultural, social and spiritual background may help women cope with mastectomy. In this background, our study demonstrates that BCS and MRM can be comparable while long-term QOL scores are considered.

Our center delivers universal health coverage and has radiation facility attached to the scheme and hence suitability of the patient to undergo breast conservation and patient’s choice were the only considerations in choosing the mode of surgery. The findings may not be generalizable to the rest of the country where availability of RT and affordability may be major concerns for choosing the treatment options. Small sample size is another limitation due to a considerable proportion of patients lost to follow-up or not willing to participate in the follow up after 5 years. Small sample size did not allow further sub analysis between the groups. The MRM patients had poor survival compared to the BCT patients in our group. This also could be due to smaller sample size, however, survival comparison is beyond the purview of this paper. Both, BCT and MRM have shown equal survival as widely accepted and demonstrated in the literature [3, 4]. The findings of this study are, however, interesting and point to that BCS may not be the golden standard in all populations, especially when resources are scarce. Larger studies from different centers in India would be required prior to establishing national guidelines regarding the standard of care for surgery for breast cancer. It points to that long term follow-up is necessary to correctly assess the outcomes after treatment for breast cancer.

## Conclusion

The QOL in patients in the present study was similar in the BCT and MRM study arms. Choice of surgical method does not appear to affect the long-term QOL scores in our cohort. Mastectomy perhaps, remains a safe and effective surgical approach in India with a comparable long-term QOL in breast cancer survivors.
